# Effects of p21 Gene Down-Regulation through RNAi on Antler Stem Cells *In Vitro*

**DOI:** 10.1371/journal.pone.0134268

**Published:** 2015-08-26

**Authors:** Qianqian Guo, Datao Wang, Zhen Liu, Chunyi Li

**Affiliations:** 1 Institute of Special Wild Economic Animals and Plants, Chinese Academy of Agricultural Sciences, Changchun, Jilin, P. R. China; 2 State Key Laboratory for Molecular Biology of Special Economic Animals, Jilin, P. R. China; St. Georges University of London, UNITED KINGDOM

## Abstract

Cell cycle is an integral part of cell proliferation, and consists mainly of four phases, G1, S, G2 and M. The p21 protein, a cyclin dependent kinase inhibitor, plays a key role in regulating cell cyclevia G1 phase control. Cells capable of epimorphic regeneration have G2/M accumulation as their distinctive feature, whilst the majority of somatic cells rest at G1 phase. To investigate the role played byp21 in antler regeneration, we studied the cell cycle distribution of antler stem cells (ASCs), via down-regulation of p21 *in vitro *using RNAi. The results showed that ASCs had high levels of p21 mRNA expression and rested at G1 phase, which was comparable to the control somatic cells. Down-regulation of p21 did not result in ASC cell cycle re-distribution toward G2/M accumulation, but DNA damage and apoptosis of the ASCs significantly increased and the process of cell aging was slowed. These findings suggest that the ASCs may have evolved to use an alternative, p21-independent cell cycle regulation mechanism. Also a unique p21-dependent inhibitory effect may control DNA damage as a protective mechanism to ensure the fast proliferating ASCs do not become dysplastic/cancerous. Understanding of the mechanism underlying the role played by p21 in the ASCs could give insight into a mammalian system where epimorphic regeneration is initiated whilst the genome stability is effectively maintained.

## Introduction

Recent advancements in regenerative biology and medicine have brought about new understandings of the mechanisms underlying organ regeneration [1]. The most dramatic organ regeneration is called epimorphic regeneration, i.e. the replacement of a lost limb/appendage from the amputation plane [2]. Current knowledge in relation to epimorphic regeneration comes predominantly from studies on lower vertebrates (i.e. amphibians), because mammals during the course of evolution, have lost the ability to fully regenerate appendages [3].

A rare exception to this rule is deer antlers, which are complex mammalian appendages, capable of complete regeneration [4–7]. Unlike the classical animal models for regeneration, which rely on cellular dedifferentiation, antler regeneration is a stem cell-based process [4,8]. A limited number of periosteal cells (estimated at 3.3 million) from the pedicle (the permanent bony protuberance, from which an antler casts and regenerates) have been identified as antler stem cells (ASCs) [4]. These ACSs are capable of achieving full antler regeneration through rapid, but well-controlled, proliferation and differentiation, with a multiplication rate comparable to/or greater than that of cancer cells [6,9,10]. However, the molecular mechanisms responsible for this regeneration are thus far unknown.

Strict control of cell cycle progression for rapidly proliferating cells is crucial to prevent them from undergoing neoplastic transformation. A typical cell cycle consists of four phases: G1 (pre-DNA synthesis), S (DNA replication), G2 (post-DNA replication) and M (cell division) [11]. To protect genomic integrity while going through the process of a cell cycle, regulatory checkpoint mechanism, are put in place [12,13]. These checkpoints ensure controlled cell cycle progression and prevent transition into subsequent phases until all processes in the previous phase are completed [14]. The first checkpoint at the G1 phase monitors DNA damage and inhibits cell cycle entry into the S phase if DNA damage is sensed. A key regulatory factor that maintains a strong G1 checkpoint is the p53-dependent p21, a cyclin dependent kinase inhibitor [15]. Once activated, p53, as the “guardian of the genome,” induces expression of its downstream target p21, which causes cell cycle arrest at G1phase, thus maintaining genomic stability in response to DNA damage induced stress [16,17]. Thus, in the absence of p21, the G1 checkpoint cannot be properly enacted [15], the cells enter S phase with possible unrepaired DNA damage, causing the cells to rely on the G2/M checkpoint, which would lead to G2/M accumulation [15,18].

MRL (Murphy Roths Large) mice, generated from cross-breeding AKR, C3H, C57BL/6 (B6), and LG strains of mice, have unique healing attributes in that they are able to close full thickness ear wounds (made with a punch) without displaying residual signs of injury or scarring [19,20]. One study reported that similar to other classical epimorphic regenerators, the fibroblast-like cells derived from the wound area in MRL mice show a distinct cell-cycle phenotype involving G2/M accumulation, and down-regulation of p21 [11,15]. Furthermore, deletion of the p21 in non-regenerative mice was enough to induce tissue regeneration similar to that observed in the MRL mice [15]. This finding implies that cell cycle checkpoint control could be a key mechanism in controlling epimorphic regeneration.

In this study we investigated whether this distinct cell cycle phenotype, G2/M accumulation and down-regulation of the p21, would also be evident in the stem cells involved in antler regeneration. Cell-cycle distribution and expression of p21 mRNA was compared in ASCs and nasal bone periosteal cells (FPCs) of a deer as the reference tissue. The role of p21 was examined using RNA interference (RNAi) to down-regulate p21 mRNA within the ASCs *in vitro*. We found that the distribution of the cell cycle in the ASCs was comparable to that of the somatic cells (FPCs), i.e. majority of cells rested at G1 phase, and no distinct G2/M accumulation phenotype was observed. This finding was in contrast to the currently held view that all types of cells capable of epimorphic regeneration have the distinct phenotype of G2/M accumulation. Interestingly, instead of down-regulation, the expression levels of the p21 mRNA in the ASCs were significantly higher than in the FPCs. Furthermore, the cell cycle of the ASCs was not affected by the down-regulation of p21 mRNA, but DNA damage and apoptosis of the ASCs increased significantly, and the process of cell aging was inhibited/slowed. The ASCs may have developed an alternative strict cell cycle control mechanism in which cell cycle re-distribution cannot be made simply by down-regulation of the p21. Therefore, the ASCs might have evolved an alternative, p21-independent G1 checkpoint mechanism. When p21 is down-regulated, this back-up mechanism may have been ignited to substitute the crippled p21-dependent checkpoint.

## Materials and Methods

### Ethics Statement

This study was approved by the Temporary Animal Ethics Committee of Institute of Special Wild Economic Animals and Plants, Chinese Academy of Agricultural Sciences (Permit Number: 2014–0035).

### Cell culture

Pedicle periosteal cells (ASCs) and facial periosteal cells (FPCs) were obtained from a two-year-old male sika deer as described previously [9] and cultured in DMEM (Life, USA) plus 10% FBS (Gibco, USA), 500 U/ml penicillin and 500 μg/ml streptomycin (Invitrogen, USA) at 37°C in 5% CO_2_. Cells were passaged using trypsin (Sigma, USA) and stored in liquid nitrogen in freezing medium (FBS +10% DMSO). When required, the ASCs and the FPCs were thawed and cultured in T75 flasks (Nest Biotechnology, USA). Cells used in this study had reached the third passage. Detailed procedures for primary ASC and FPC culture have been described elsewhere [21].

### Cell-cycle analysis

Cell cycle distribution in the ASCs and FPCs were assayed by flow cytometry. Cells were initially seeded at the density of 3x10^4^ cells/cm^2^ and the assay was conducted at cell exponential growth phase. Briefly, cells were trypsinized, washed twice in 10 ml pre-cooled PBS (phosphate buffer solution), fixed in 75% ethyl alcohol at -20°C overnight, centrifuged at 500g, and resuspended in 500μl PBS containing 10μl RNase (50 μg/ml; Sigma, USA) for 30min at 37°C. Cells (1×10^6^/experiment) were labeled with 50μl PI (Propidium Iodide, final concentration: 50μg/ml, Shanghai Shenggong Inc, China) for 30 min in the dark. Cell cycle staging was then analyzed using a flow cytometer (Multi SET Software for acquisition and ModFit LT Software for analysis; channel: FL2, CV≤8%; BD FACSCalibur, USA).

### RT-qPCR

Total RNA was isolated from cells using Trizol extraction and purified on a silica base spin column (SK1321, Shanghai Shenggong Inc, China) according to manufacturer’s protocol. RNA was resuspended in RNase-free water and RNA concentration and purity was assessed using a NanoVue (GE, USA). Reverse transcription and qPCR were performed to determine expression levels of p21 mRNA. Each pair of PCR primers (Table 1) were designed to span an exon boundary to avoid amplification of genomic DNA [22]. PCR products of the expected size were detected on an agarose gel, and the efficiency of each primer set for RT-qPCR was determined to be almost 100%. Briefly, total RNA (2μg) was reverse-transcribed to cDNA using a Primescript 1^st^ Strand cDNA Synthesis Kit (TaKaRa, Dalian, China). qPCR was performed using an ABI PRISM7500 qPCR System (ABI, USA) with FastStart Universal SYBR Green Master (ROX) (Roche, USA). Reactions contained 13μlSYBR GreenPCR Master Mix, 300 nM of each primer (Table 2), and 1μl of cDNA in a total volume of 25 μl. PCR conditions were 95°C for 15s, followed by 35 cycles of 94°C for 15s, 57°C for 30s, and 72°C for 32s. Melting curve analysis was conducted to verify specificity of the PCR. Each reaction was run in triplicates. β-Actin gene expression was used for normalization. The relative expression level of the p21 mRNA was analyzed using 7500 System Software. The control was normalised to 1 and expression data were presented as bar graphs.

**Table 1:** Primers used for qPCR.

PCR/ gene	Forward	Reverse
qRT-PCR/ p21 gene	GACCACTTGGACCTGTCGCT	GGGTTAGGGCTTCCTCTTGG
qRT-PCR/β-actin gene	GCGTGACATCAAGGAGAAGC	GGAAGGACGGCTGGAAGA

**Table 2:** Interfering sequences of siRNA targeting for p21 gene.

group	siRNA	loop
S1-sense	5’-cgcgtcccc GCGGTGGAACTTCGACTTT ttcaagaga AAAGTCGAAGTTCCACCGC tttttggaaat-3’	ttcaagaga
S1-antisense	5’-cgatttccaaaaaGCGGTGGAACTTCGACTTT tctcttgaa AAAGTCGAAGTTCCACCGC gggga-3’	tctcttgaa
S2-sense	5’-cgcgtcccc CCAGCATGACAGATTTCTA ttcaagaga TAGAAATCTGTCATGCTGG tttttggaaat-3’	ttcaagaga
S2-antisense	5’-cgatttccaaaaaCCAGCATGACAGATTTCTA tctcttgaa TAGAAATCTGTCATGCTGG gggga-3’	tctcttgaa

### siRNA synthesis for deer p21

Based on the sequence of the sika deer *p21* gene (in submission), two siRNA sequences targeting the deer *p21* gene were designed using Angela siRNA design rules and the MIT online tool (http://jura.wi.mit.edu/bioc/siRNAext/home.php); these were subsequently designated as S1 and S2 (Table 2). These two candidates were blasted against the NCBIRefSeq RNA database(http://blast.ncbi.nlm.nih.gov/Blast.cgi) to confirm their specificity, and empirically annotated to form oligonucleotides of shRNA (short hairpin RNA) prior to synthesis (Shanghai Shenggong Inc, China). The synthesized oligonucleotides were subsequently annealed into double stranded small hairpin RNAs.

### Construction of lentiviral siRNA vector

The lentiviral vector system (gift from Prof. George Liu, Beijing University [23]), consisting of pLVTHM, pCMV and pMD2G plasmids, was used to deliver shRNA into the ASCs in this study. The plasmid pLVTHM contains a human H1 promoter which can sustain expression of a shRNA and GFP (Green Fluorescent Protein). Each shRNA sequence, S1 or S2, was inserted into the site between Cla1 and Mlu1 of the pLVTHM plasmid. The pMD2G plasmid includes the VSV-G gene which provides the capsid protein for virus packaging, and the pCMV plasmid encodes the necessary viral constitutive genes. Each shRNA sequence was ligated into the pLVTHM plasmid using T4 ligase (Thermo, USA). The recombinant DNA (pLVTHM-siRNA) or empty carrier (pLVTHM as negative control), pCMV and pMD2G were co-transfected into 293T cells using lipofectamine 2000 reagent (Invitrogen, USA) according to manufacturer’s protocol. Virus-containing supernatants were collected 24h and 48h after transfection respectively, pooled together, then concentrated by centrifugation using the Amicon ultra centrifugal filter devices (Millipore Corporation, USA), and stored at -80°C.

### Lentiviral infection

ASCs at the third passage were seeded in a 6-well culture plate (Corning Coster, NY, USA) and upon reaching 50% confluence, the ASCs were infected. Briefly, the medium was removed and replaced with lentiviral-vector supernatants (S1, S2, or empty carrier respectively) or with the normal culture medium (an additional control) in the presence of 8μg/ml polybrene (Sigma, USA). Forty eight hours after infection the monitoring of GFP expression was initiated, using a fluorescent microscope (Leica, Germany), to determine the levels of siRNA expression. The GFP expressing cells were sorted by flow cytometry (BD FACSAria, USA) according to the manufacturer’s manual.

### Proliferation Assay

The proliferation rate of the ASCs was measured at the sixth and fifteenth passages, using a MTT assay as previously described [24]. In brief, cells at the logarithmic growth phase were seeded in triplicates into 96-well plates at a density of 5000 cells/well and cultured for 1–6 days. At each time point, cells were incubated in medium containing 20μl MTT/well for 4 hours. Dimethyl sulfoxide (150μl; DMSO, Sigma, USA) was added to solubilize the formazan crystals and the OD_595_ measured on an ELISA plate reader (Tecan, Switzerland).

### Apoptosis of cells

Apoptosis was detected using Annexin V-PE/7-AAD staining (Apoptosis Detection Kit; KGA 1017 Kaiji Inc, Nanjing, China). Briefly, 1–2×10^6^ cells were trypsinized using EDTA-free trypsin (Invitrogen, USA) and centrifuged at 2000 rpm, washed twice in 10 ml PBS, then labeled with 7-AAD and Annexin V-PE in binding buffer according to manufacturer's instructions. To identify the apoptotic population of ASCs, fluorescent signals were detected with flow cytometry (channels: FL2/FL3, BD FACSCalibur, USA).

### Comet assay for the detection of DNA damage

DNA damage in the ASCs was detected using an alkaline comet assay (alkaline single-cell gel electrophoresis assay; Cleaver, Britain), following the protocol previously described [25,26]. Briefly, a cell suspension (where cell viability was over 95% using trypan blue exclusion analysis) was mixed with 0.6% low-melting-point agarose (kept at 37°C), then rapidly spread onto specially treated slides (4250-050-K, Trevigen, USA) and covered with a 24x24 mm cover slip. After immobilizing at 4°C for 15 minutes, the slide was submerged in precooled lysis solution (2.5 M NaCl, 30 mM Na_2_EDTA·2H_2_O, 10 mM Tris, and 1% Triton X-100) for 1.5h at 4°C in the dark. The slides were then placed in electrophoresis solution (900 mM Tris, 900 mM H_2_BO3, 20 mM Na_2_EDTA·2H_2_O) for 20 minutes to facilitate DNA unwinding. Electrophoresis was conducted for 30 minutes at 20 volts. After electrophoresis the slides were stained with ethidium bromide (5μg/mL) and comets were visualized under a fluorescent microscope (Leica, Germany) at 100× magnification. The degree of DNA damage was assessed using the tail lengths, tail DNA% and Olive tail moment, which were calculated from 100 randomly chosen cells per group with the CASP software (Comet Assay Software Project).

### Senescence-associated β-galactosidase staining

ASC senescence was assessed using a Senescence β-galactosidase Staining Kit (Biyuntian Inc, china). Briefly, cells at the fifteenth passage were seeded in 6-well plates at a density of 4×10^4^ cells/cm^2^, and cultured at 37°C, 5% CO_2_ for 24h, prior to staining. The aging cells were stained for β-galactosidase according to the manufacturer’s protocol (deep blue). Positive cells were observed under a microscope (Olympus, Japan), and the mean percentage of cells expressing β-galactosidase was calculated. The experiment was repeated three times and the results were analyzed statistically.

### Statistical analysis

All experiments were performed in triplicates and three independent experiments were conducted. Results are presented as the mean ± SEM of three independent experiments. Statistical analysis was performed with t test using SPSS (windows version 20) and values at p< 0. 05 were considered statistically significant.

## Results

### Cell cycle in the ASCs and the FPCs

**Fig 1:**
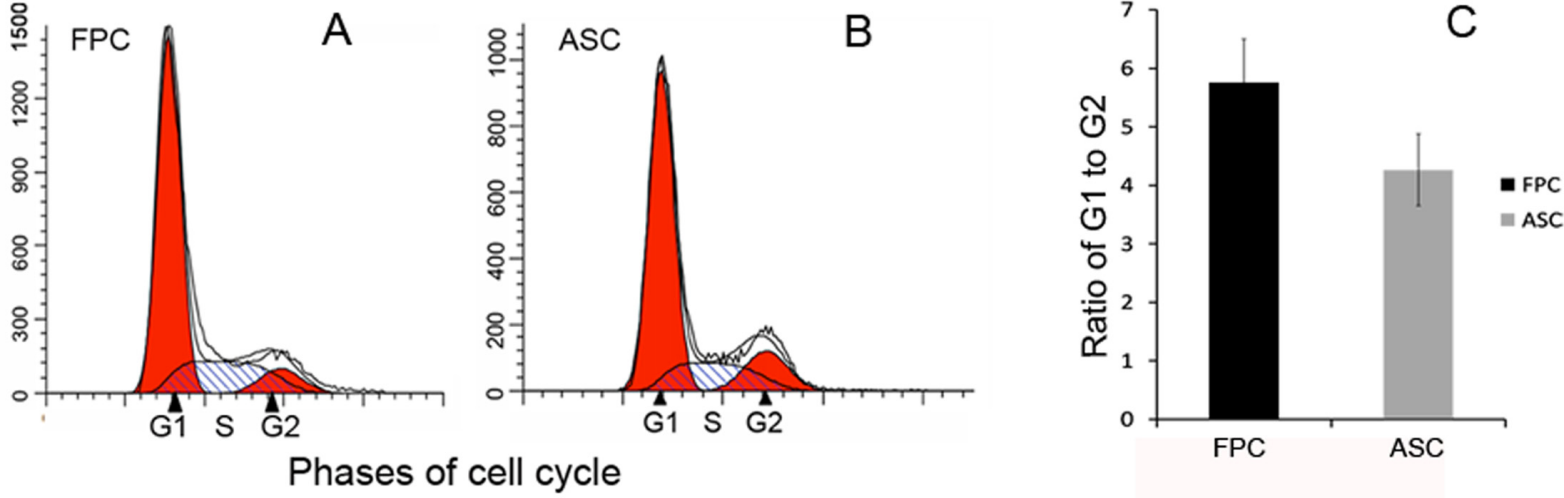
Cell cyclein the ASCs and the FPCs. Exponentially growing ASCs and FPCs were harvested for the cell cycle analysis using flow cytometry. The data were analyzed with ModFit LT Software to estimate the percentages of cells at G1, S, and G2/M phases. A: Cell cycle distribution of the FPCs. B: Cell cycle distribution of the ASCs. No distinct G2/M accumulation was observed in either of the cell lines. C: Ratio of the cell numbers at G1 to G2 phase. Note that there was no significant difference in cell cycle distribution between these two cell types (P>0. 05).

As described previously, G2/M cell cycle accumulation is commonly reported in regenerative organisms [15]. To ascertain whether the ASCs capable of epimorphic regeneration, also express this distinct feature, the cell cycle of the ASCs and the FPCs (as a reference cell line), at the sixth passage were analyzed using flow cytometry. Cell cycle distributions of the ASCs and the FPCs at the exponential growth phase are shown in Fig 1 and the actual percentages of cells in G1, S and G2/M are shown in Table 3. The majority cells in both the FPCs (Fig 1A) and the ASCs (Fig 1B) rested at the G1 phase and no distinct G2 accumulation was observed for these cell types. There was no significant difference in the ratio of the cell numbers at G1 to G2 phases between the ASCs and the FPCs (Fig 1C, P = 0.192). These results indicate that the cell cycle of the ASCs is comparable to the control somatic cells (FPCs).

**Table 3:** Cell cycle distribution of the FPCs and the ASCs.

Group	G0/G1	S	G2/M
FPC	63.9±3.74	25.1±3.42	11±1.03
ASC	59.9±4.54	25.9±3.07	14.2±0.64

### Expression levels of p21 mRNA in the ASCs and the FPCs

**Fig 2:**
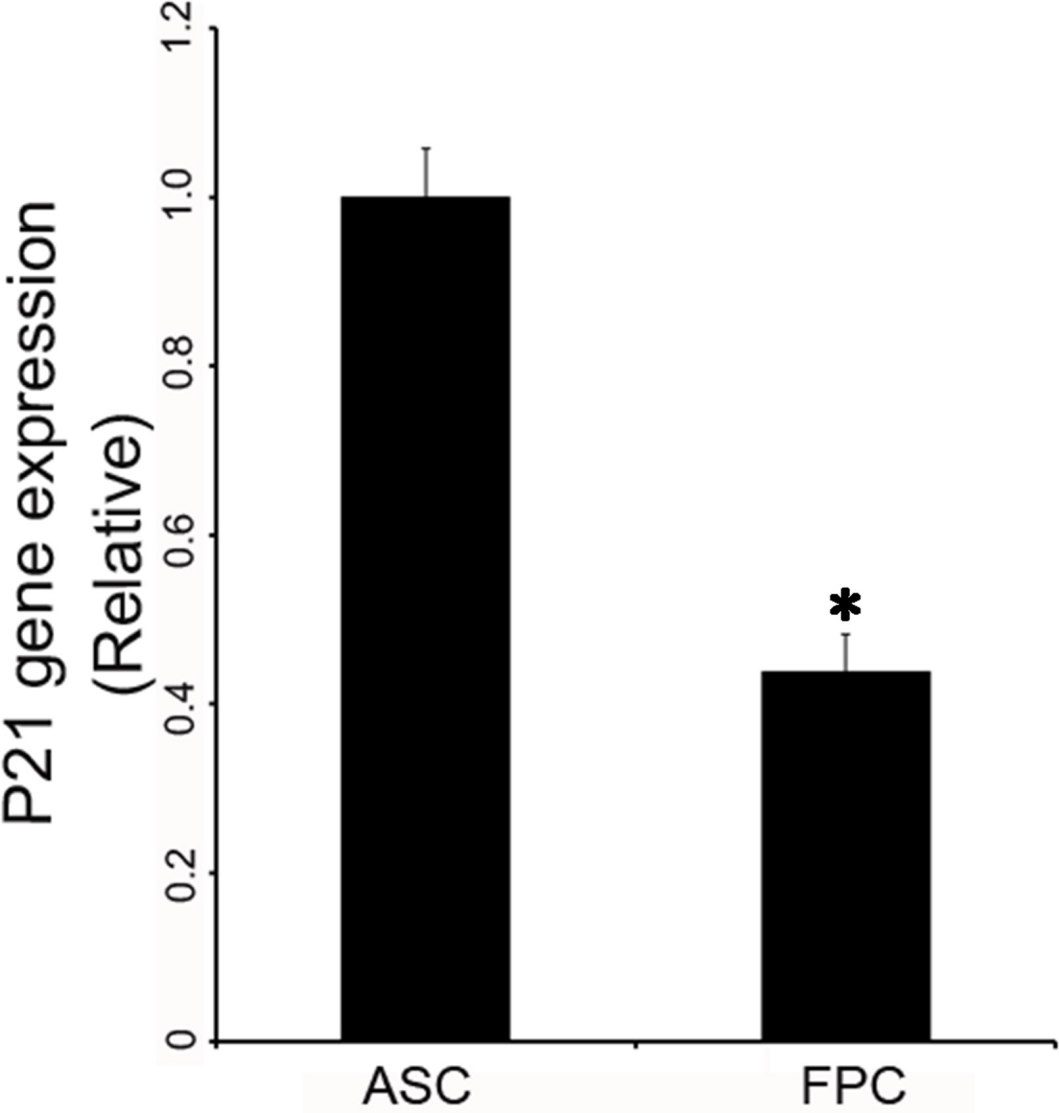
Expression levels of p21 mRNA in the ASCs and the FPCs. Total RNA was isolated from the ASCs or the FPCs at the third passage, RT-qPCR was performed for each cell type. Note that mRNA level for p21 in the FPCs accounted for only half of that in the ASCs. Expression level of the p21 mRNA in the ASCs was significantly higher than that in the FPCs (P<0.05).

In the mouse model for regeneration, MRL cells express lower levels of p21 than control mouse cells [15]. To learn if there was also a difference in this aspect between the ASCs and the FPCs, expression levels of p21 mRNA from cells at the third passage was measured. As shown in Fig 2, expression levels of p21 mRNA in the FPCs accounted for only half of that in the ASCs. In other words, instead of down-regulation, p21 mRNA in the ASCs was significantly higher than that in the control FPCs (P = 0.003). This is in sharp contrast to the results from the mouse model for regeneration. To determine whether the difference in cell cycle distribution between the ASCs and the cells from the other classical epimorphic regenerators was due to the high expression levels of p21, down-regulation of p21 in the ASCs was carried out using RNAi technology.

### Effects of RNAi on the p21 expression in the ASCs

**Fig 3:**
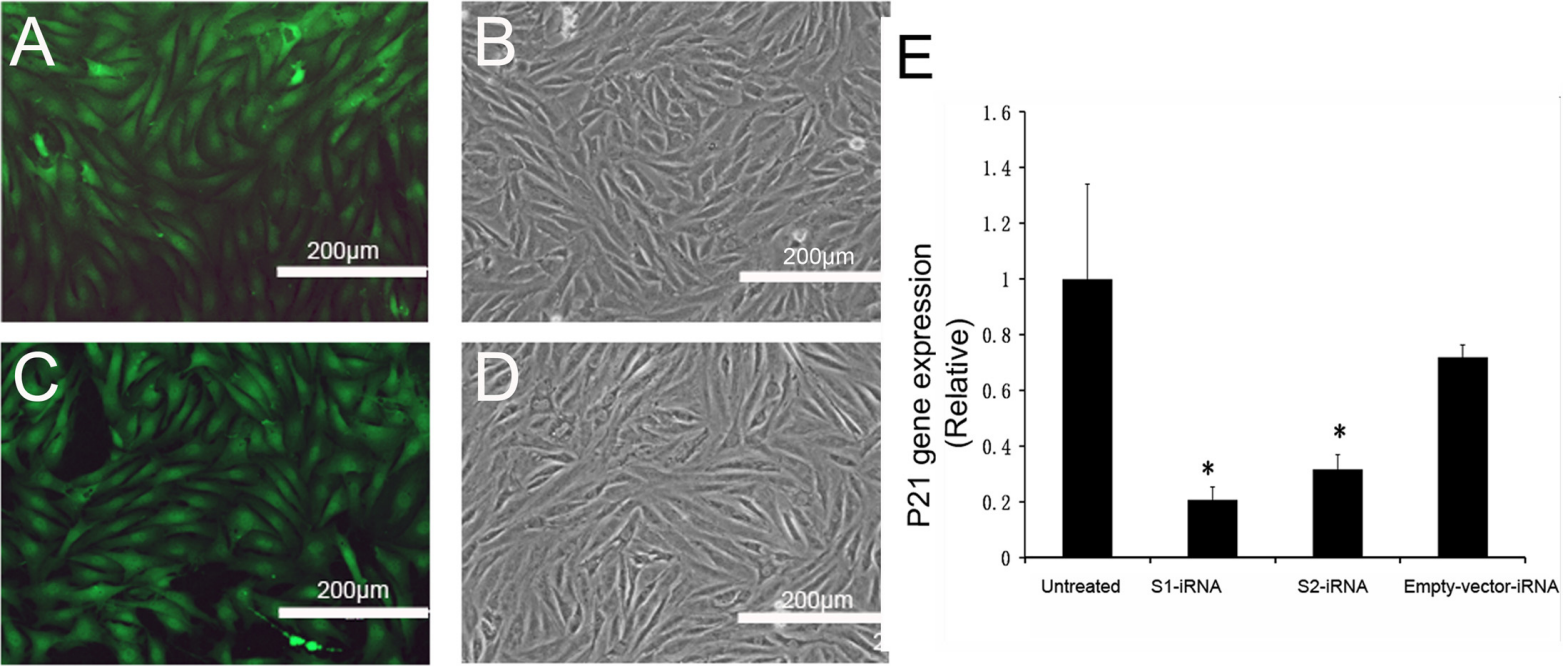
Effects of RNAi on the p21 expression in the ASCs. GFP expression was monitored under a fluorescent microscope 48h after lentiviral infection. A and B, the p21-shRNA-infected ASCs. C and D, the empty-vector-infected ASCs. Fluorescence was clearly observable in both the p21-shRNA-infected ASCs (3A) and the empty-vector-infected ASCs (3C). Cells from both groups were healthy. E: RT-qPCR of the p21 expression. Note that both of the target sequences (S1 and S2) significantly down-regulated p21 mRNA expression in the ASCs (P<0.05). No significant difference was found between the empty-vector-iRNA ASCs and the untreated ASCs (P>0.05).

To determine the efficiency of viral infection, the ASCs infected with either target shRNA or empty carrier were examined 48h after infection. Fluorescence was clearly observable in both the shRNA-infected ASCs (Fig 3A) and the empty-vector-infected ASCs (Fig 3C). The cells from both the shRNA-infected and the empty-vector-infected all appeared to be healthy (Fig 3B and 3D). These results confirmed that the lentiviral infection was successful.

Subsequently, qPCR was performed to determine the effects of RNAi on expression levels of p21 mRNA (Fig 3E). Total RNA was isolated from the lentiviral-infected or uninfected ASCs 48hrs after infection. Results showed that both the target sequences (S1 and S2) significantly down-regulated the p21 expression in the ASCs compared to the untreated ASCs (S1: P = 0.001; S2: P = 0.002); and the S1 target sequence (knockdown rate: 79.4%) was more efficient at down-regulating expression than S2 (knockdown rate: 68.4%). In contrast, no significant difference was found between the empty-vector-iRNA ASCs and the untreated ASCs (P = 0.098). We therefore selected the S1target sequence for the following studies.

### Effects of p21 knockdown on ASC cell-cycle distribution and ASC growth

**Fig 4:**
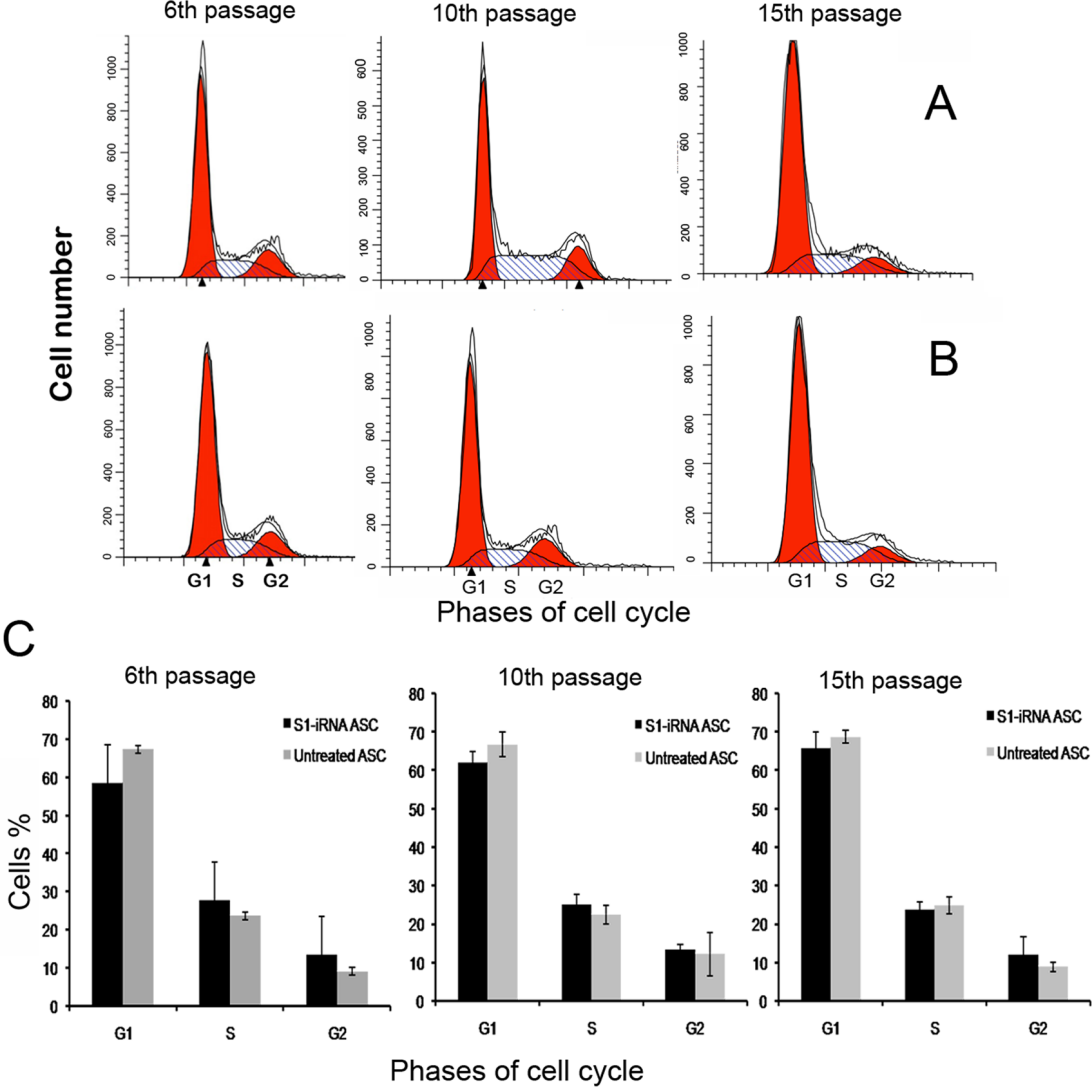
Effects of p21 knockdown on ASC cell-cycle distribution. Cells were infected at the third passage and harvested at the sixth, tenth and fifteenth passages, and analyzed by flow cytometry. The data were analyzed with ModFit LT Software to estimate the percentages of cells at G1, S, and G2/M. A and B: Cell cycle distribution of the ASCs. A, S1-iRNA ASCs. B, Untreated ASCs. Note that there was no distinct G2 accumulation in either of the groups. C: Bar graph depicts percentage of the cells at G1, S and G2 phases. Percentage of the S1-iRNA ASCs at G2 phase was similar to that of the untreated ASCs. There was no significant difference in cell-cycle distribution between the two groups (P>0. 05).

The cell cycle of ASCs was comparable to that of the control cells (FPCs), which is different from the other regenerators (G2/M accumulation). To investigate the effects of p21 knockdown on the ASC cell-cycle distribution, the untreated ASCs and the S1-iRNA ASCs, at the exponential growth phase, were analyzed by flow cytometry. Cells were infected by lentivirus at the third passage and harvested at the sixth, tenth and fifteenth passages for cell cycle distribution analysis. The results showed that no significant changes in cell cycle distribution were observed for the S1-iRNA ASCs (Fig 4A) compared to the untreated ASCs (Fig 4B) at the passages examined. Percentages of cells at G1, S and G2/M phases did not show any significant difference (Fig 4C). Down-regulation of p21 did not cause ASC cell-cycle re-distribution toward G2/M accumulation.

**Fig 5:**
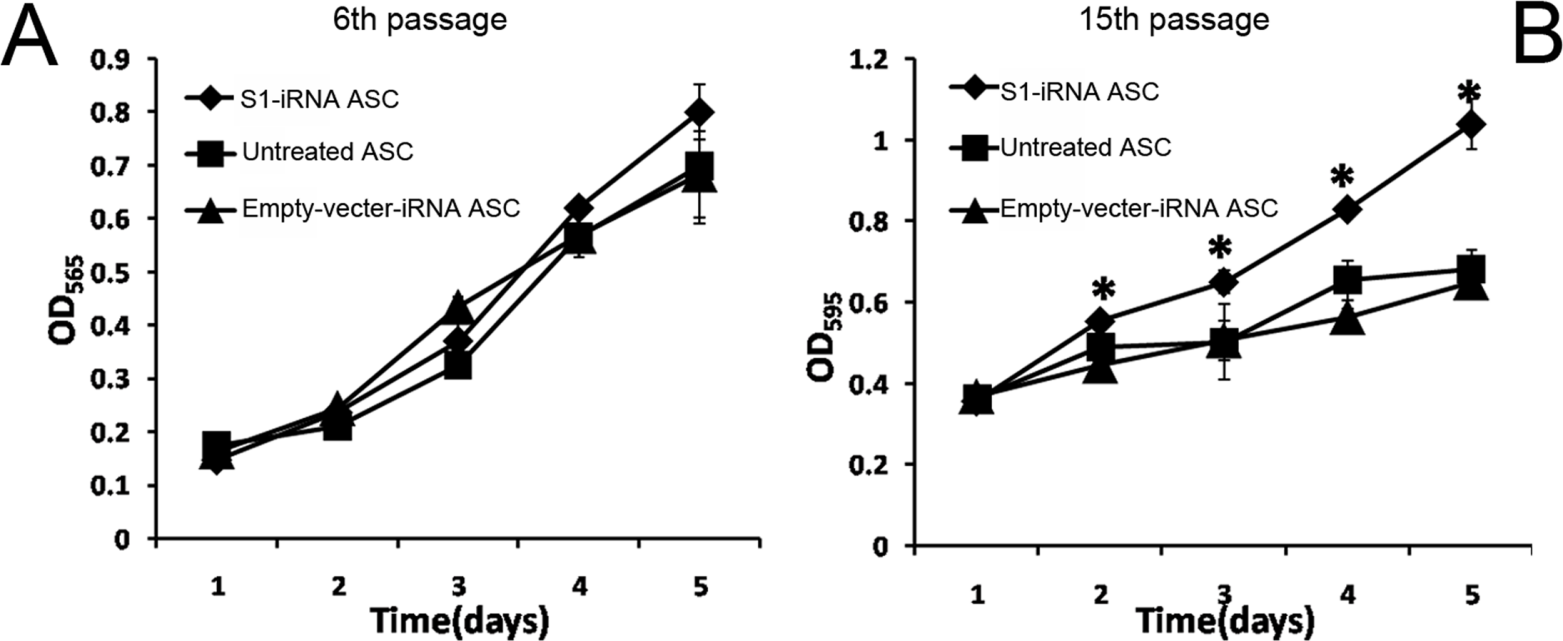
Effects of p21 knockdown on ASC growth. MTT assay of the S1-iRNA ASCs, the empty-vector-iRNA ASCs and the untreated ASCs. A, Growth curve of the ASCs at passage six. No significant difference was observed between these three groups. B, Growth curve of the ASCs at passage fifteen. Note that the proliferation rate of the S1-iRNA ASCs was significantly higher than those of the other two groups (P<0.05).

To investigate the effects of down-regulation of p21 on the ASC proliferation, growth rates of the S1-iRNA ASCs, the empty-vector-iRNA ASCs and the untreated ASCs were conducted at the sixth and the fifteenth passages using an MTT assay. As shown in Fig 5, there was no significant difference between the S1-iRNA ASCs, the empty-vector-iRNA ASCs and the untreated ASCs at the sixth passage (Fig 5A). The growth rate of the ASCs was lower at later passages than at earlier passages as determined by the gradient of the growth curves. At passage fifteen the proliferation rate of the S1-iRNA ASCs was, however, significantly higher than that of the untreated and the empty-vector-iRNA ASCs (Fig 5B), which indicates that knockdown of the p21 delayed this decrease in growth rate of the ASCs.

### Effects of p21 knockdown on DNA damage in the ASCs

**Fig 6:**
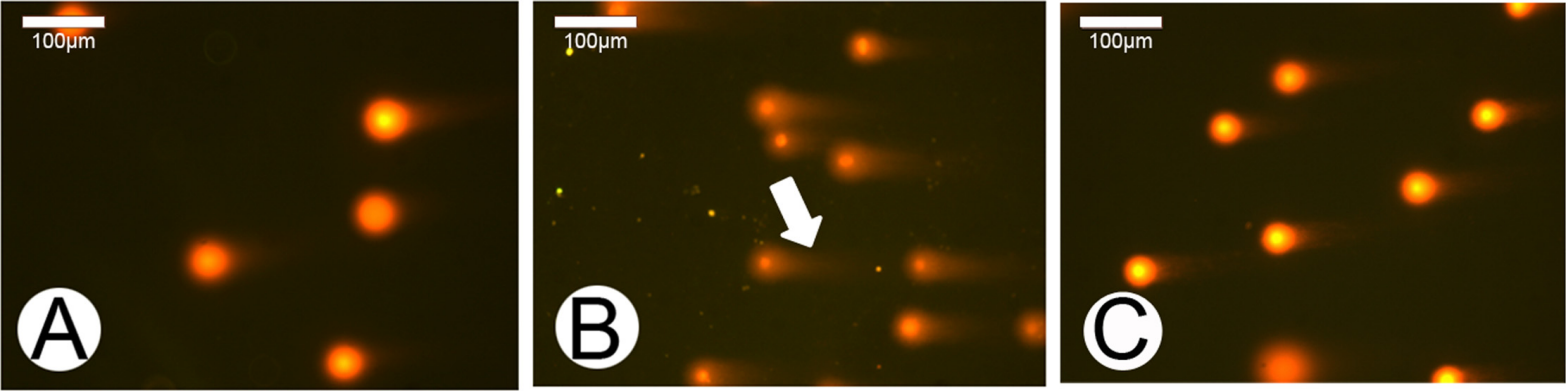
Effects of p21 knockdown on DNA damage in the ASCs. Observation of the cells containing DNA breaks (with long tails) under a fluorescent microscopy. A, Untreated ASCs. No obvious comet tails were observed. B, S1-iRNA ASCs. Almost every cell in the field had a long and obvious comet tail (arrow). C, Empty-vector-iRNA ASCs. Weak comet tails were observed.

To determine the effects of p21 knockdown on DNA damage in the ASCs, alkaline comet assays were performed on the S1-iRNA ASCs, the empty-vector-iRNA ASCs and the untreated ASCs at passage ten after 48h of treatment/control conditions. The majority of the untreated ASCs were devoid of fluorescent tails (Fig 6A), indicating that nuclear DNA of these cells remained intact. A few empty-vector-iRNA ASCs showed weak comet tails (Fig 6C). In contrast, down-regulation of p21 in the ASCs (treated group) increased the number of comets with typical tails (Fig 6B). As shown in Table 4, suppression of p21 expression in the ASCs lead to a significant increase in comet tail length (48.8±2.74) compared to the empty-vector-iRNA ASCs (24.96±1.15, P = 0.00001) and the untreated ASCs (18.16±1.02, P = 0.00001). Consistence with these results, comet tail DNA% and Olive tail moment in the S1-iRNA ASCs (22.68±3.16, 7.33±0.76, respectively) were both significantly higher than those of the empty-vector-iRNA ASCs (11.06±1.13%, 2.43±0.14, respectively) and the untreated ASCs (7.07±0.69%, 1.88±0.19, respectively). These data indicate that the p21 knockdown has deleterious effects on DNA stability in the ASCs.

**Table 4:** Effects of p21 knockdown on the ASCs DNA damage.

Group	Tail Length	Tail DNA%	Olive Tail Moment
UN	18.16±1.02	7.07±0.69	1.88±0.19
S1-iRNA	48.8±2.74**	22.68±3.16*	7.33±0.76*
Em-iRNA	24.96±1.15*	11.06±1.13	2.43±0.14

UN: The untreated ASCs; S1-iRNA: The S1-iRNA ASCs; Em-iRNA: The empty-vector-iRNA ASCs.

** p<0.01

* p<0.05

### Effects of p21 knockdown on apoptosis of the ASCs

**Fig 7:**
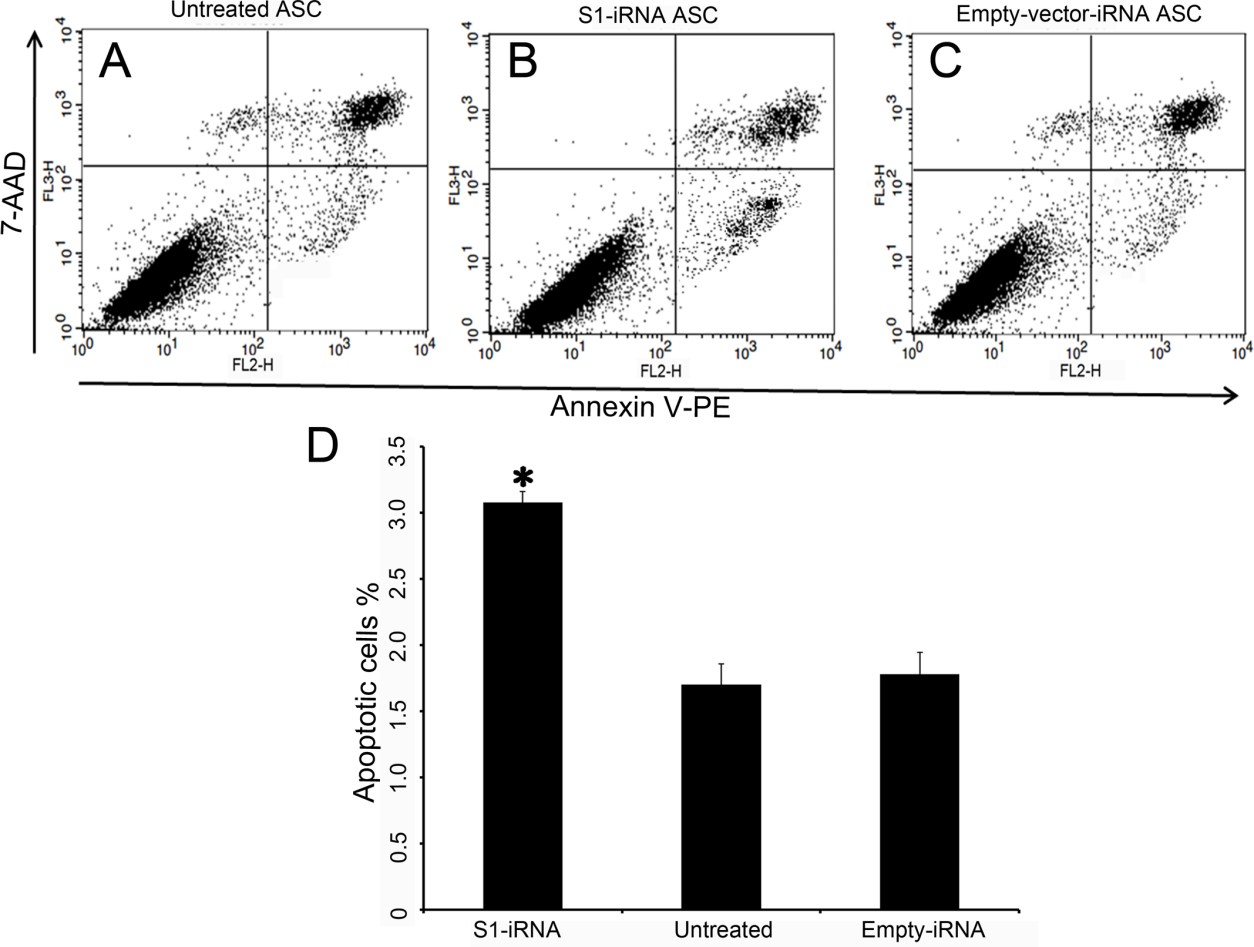
Effects of p21 knockdown on apoptosis of the ASCs. Detection of cell apoptosis by Annexin V-PE/7-AAD staining. A-C: Annexin V-PE was read in FL2 and plotted on the x-axis. 7-AAD was read in FL3 and plotted on the y-axis. Living cells were located in the lower left quarter (both annexin and 7AAD negative), apoptotic cells were in the lower right quarter (annexin positive, 7AAD negative) and the dead cells (and late apoptosis) were in the top right quarter (both annexin and 7AAD positive). A, Untreated ASCs. B, S1-iRNA ASCs. C, Empty-vector-iRNA ASCs. D, Comparisons of the apoptotic rate between the S1-iRNA ASCs, the empty-vector-iRNA ASCs and the untreated ASCs. Note that the rate of apoptosis in the S1-iRNA ASCs was significantly increased compared to the untreated ASCs and the empty-vector-iRNA ASCs (P<0.05).

We also investigated the effects of the p21 knockdown on cell apoptosis using an Annexin V-PE/7-AAD Apoptosis Detection Kit. Cells were infected with lentivirus at the third passage. The infected cells were harvested at the fifth passage for the detection of apoptosis. In Fig 7, apoptotic S1-iRNA ASCs were significantly increased compared to the untreated ASCs (P = 0.000381) and the empty-vector-iRNA ASCs (P = 0.000535), while no significant difference was detected between the untreated ASCs and the empty-vector-iRNA ASCs (P = 0.681). Down-regulation of p21 resulted in an increase of apoptotic cells 3.08±0.078%, whereas only 1.78±0.162% and 1.7±0.154% in the empty-vector-iRNA ASCs and the untreated ASCs were detected respectively.

### Effects of p21 knockdown on senescence in the ASCs

**Fig 8:**
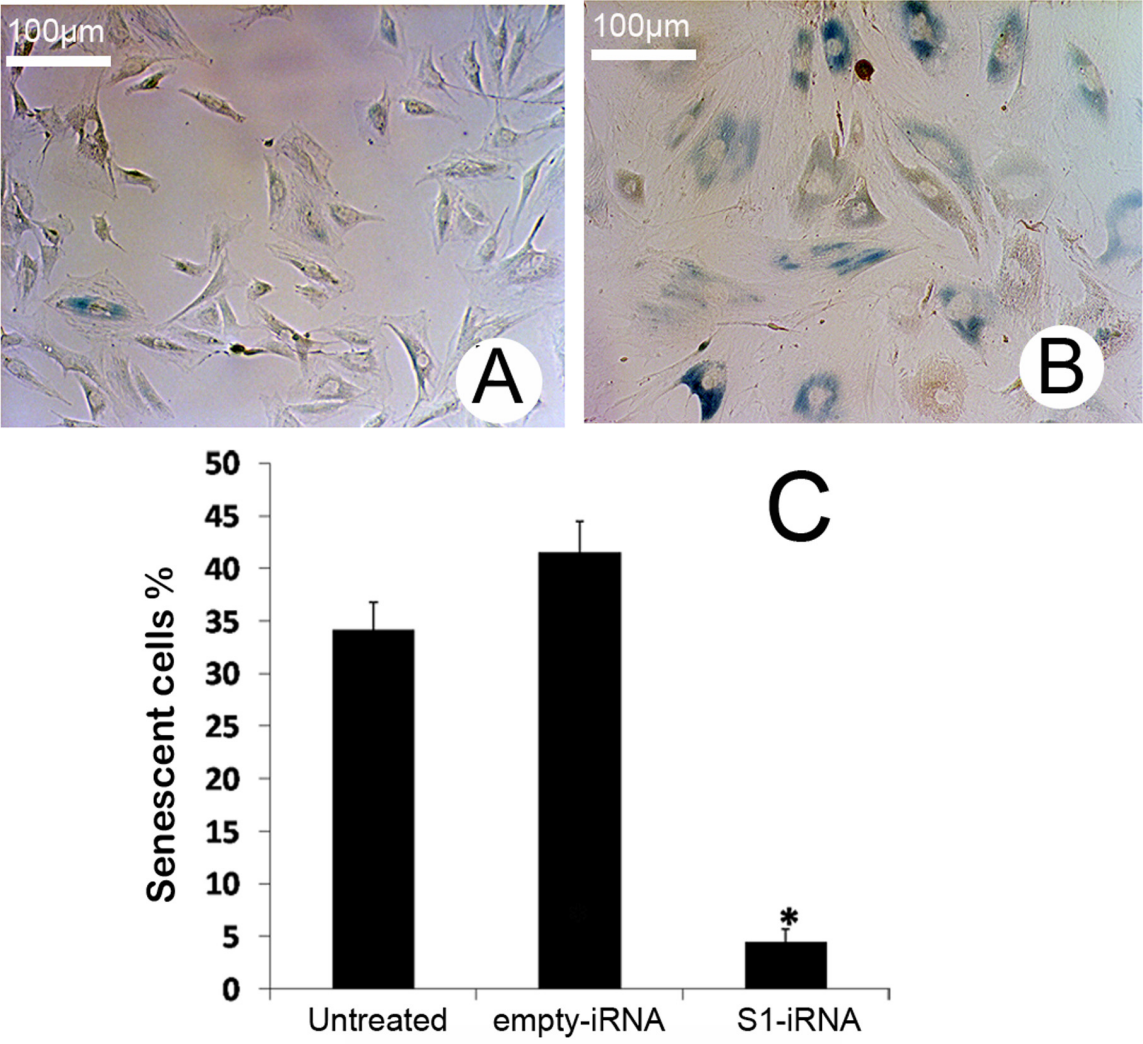
Effects of p21 knockdown on senescence in the ASCs. A and B: β-Galactosidase staining. A, S1-iRNA ASCs. Note these cells were barely stained, changes in cell volume were not detectable. B, Empty-vector-iRNA ASCs. Note most of these cells were stained in deep blue (senescent cells), with an increase in cell volume. C, Comparisons between the S1-iRNA ASCs, the empty-vector-iRNA ASCs and the untreated ASCs in the percentage of the senescence positive ratio of the cells. Note that senescent rate of the cells infected by S1-siRNA was significantly lower than those of the other two groups (P<0.05).

Based on the function of p21 in cell senescence, we investigated the effects of p21 knockdown on senescence of the ASCs by a Senescence β-galactosidase Staining Kit. As the results shown, at passage fifteen the S1-iRNA ASCs were barely expressing senescence-associated β-galactosidase (Fig 8A). In sharp contrast, the percentage of cells expressing β-galactosidase increased in the empty-vector-iRNA ASCs, which indicates that these cells were becoming senescent (Fig 8B). With careful comparison, we also detected that the size of the empty-vector-iRNA ASCs was increased. As shown in Fig 8C, the percentage of aging ASCs following *p21* gene knockdown (4.48±1.13%) was significantly decreased compared to the empty-vector-iRNA ASCs (41.52±2.95%, P = 0.000118) and the untreated ASCs (34.20±2.56%, P = 0.000672). In contrast, there was no significant difference in aging cell number between the untreated ASCs and the empty-vector-iRNA ASCs (P = 0.053). The results suggest that the p21 plays an important role in the ASC senescence.

## Discussion

To the best of our knowledge, this study is the first to examine cell-cycle regulation in relation to antler regeneration. As a key regulatory factor at the G1 checkpoint of the cell cycle, p21 has been confirmed to play an important role in cell-cycle distribution and tissue regeneration [15]. Unexpectedly, in the present study, our results showed that down-regulation of p21 did not alter cell-cycle distribution of the ASCs toward G2/M accumulation, suggesting that an alternative regulatory mechanism may have been activated when p21was down-regulated. However, DNA damage and apoptosis of the ASCs significantly increased and the cell aging process significantly slowed following down-regulation of p21. Therefore, in the ASCs p21 protein may contribute to the regulation of DNA damage repair, which is crucial to the maintenance of genome stability, in order to prevent such fast proliferating cells from undergoing neoplastic changes.

Cell cycling is an integral part of cell proliferation, and the latter is essential for tissue/organ regeneration. A typical cell cycle consists of four phases, G1, S, G2 and M, these phases take place sequentially when a cell is activated to divide from its quiescent state, termed G0[11]. Checkpoints exist in these phases to prevent the cells from entering S and M phases precociously until all requirements for DNA synthesis and mitosis are satisfied, in so doing reducing the risk of cells becoming malignant. If cells sense DNA damage or other stresses during the G1 phase, cell cycle progression would be arrested. If the G1 checkpoint fails, it would lead to dependence on the G2 checkpoint [27]. Interestingly, G2/Maccumulation is reportedly a common feature in regenerating organisms [15], such as hydra [28], amphibian [29], mammalian liver [30, 31] and MRL mouse ear [32]. In these animal models, cells derived from regenerative tissues escape the G1 checkpoint to reach the next checkpoint, resulting in G2/M accumulation. An advantage of the G2/M accumulation is the increase in cell proliferation rate, which is needed for regeneration. Nonetheless, it also involves the risk of uncontrolled proliferation as the G2/M phase is the last checkpoint of a cell cycle. Notably, the ASCs that possess the full potential of epimorphic regeneration [7] exhibit a cell cycle that is comparable to normal somatic cells (i.e. FPCs), i.e. the majority of cells rest at the G1 phase instead of G2/M. Therefore, deer antlers not only provide a single model for mammalian epimorphic regeneration, but also might be a unique model for risk-free epimorphic regeneration.

Previous studies have shown that p21 is involved in the regulation of the cell cycle as a major controlling factor at the G1 checkpoint. The *p21* gene is the major downstream target gene of p53 [11]. Before a cell enters S-phase, DNA damage, oxidative stress, or other DNA damaging factors can activate the expression of p53, and the targeted p21 will then inhibit the activity of cyclins at the G1 checkpoint, the consequence of which is to stop the transition from G1 phase to S phase [33]. Generally, a typical cell cycle stops at the G1 checkpoint [11]. However, cells capable of epimorphic regeneration skip the G1 checkpoint and are arrested at the G2/M phase [11]. Therefore, the G2/M phase accumulation has been known as the distinct cell cycle phenotype for epimorphic regeneration [15]. Bedelbaeva et al [15] reported that deletion of the *p21* gene induced ordinary mouse to acquire a regenerative phenotype and the resultant cells participating in regeneration expressed the distinct cell-cycle involving G2/M accumulation. Therefore, p21 must be crucial for initiating the process of regeneration possibly via regulation of the G1 checkpoint. However, our results showed that down-regulation of the p21 did not alter cell-cycle of the ASCs toward G2/M accumulation, which is different to the results found in the mouse model of regeneration. This finding suggests that the ASCs may have developed an alternative strict cell cycle control mechanism in which cell cycle re-distribution could not be made simply by down-regulation of p21. This mechanism may have been activated as a substitution to resist the alteration of cell cycle distribution when p21 in the ASCs is down-regulated.

In addition to the role in cell cycle regulation p21 is also involved in other cellular functions, such as DNA damage repair and apoptosis. The DNA of rapidly growing cells is more inclined to damage due to both external and internal stresses [34]. During cellular reprogramming, DNA damage at a certain level is tolerated, if the damage exceeds that upper limit, the damage would trigger cell apoptosis [35,36], or the faulty copies of DNA will eventually lead to neoplastic transformation [37,38]. Perucca et al [39] reported that expression of p21 is closely associated with DNA damage repair and apoptosis, as loss of the p21 activates a DNA damage response in normal human fibroblasts. It is also reported that p21 raises the threshold of apoptosis induced by DNA-damage in prostate cancer cells [40]. In this research down-regulation of the p21 resulted in cell apoptosis and substantial DNA damage in the ASCs. These findings suggest that p21 may play a role in the resistance to apoptosis and participate in DNA damage repair in the ASCs. Given the significant role of DNA damage repair processes in protecting against cancer [41], future work in the ASCs along this line should be aimed at elucidating whether p21 is involved in the maintenance of genome stability by modulating DNA repair processes.

Cells inevitably will become senescent, and p21 plays a role in this process. Nuclear accumulation of p21 protein in human colonic carcinoma cells is found to cause cellular senescence [42]. Loss of p21 function results in an extension of cell lifespan both *in vitro* and *in vivo*[43,44], and causes cells to bypass telomere-dependent replicative and oncogene-induced senescence in normal human fibroblasts and MEFs [45]. Consistence with these reports, in the present study, down-regulation of the p21 slowed the process of senescence of the ASCs, indicating that p21 indeed contributes to the process in which rapid proliferation of the ASCs is precisely regulated without undergoing neoplastic transformation. Given the importance of genome stability to a healthy cell line, we speculate that p21 may be involved in maintaining genome stability in the ASCs, which is crucial for the cells capable of rapid regeneration, though this will require future study.

Overall, the ASCs did not show distinct G2 accumulation, and hence deer antlers not only provide a single model for mammalian epimorphic regeneration, but also may be a unique model for risk-free epimorphic regeneration. The discovery of a possible function of p21 in maintaining genome stability in the ASCs would give insight into a system where epimorphic regeneration is enacted whilst the genome stability is effectively maintained.

## References

[1] Stocum DL. Regenerative biology and medicine. J Musculoskelet Neuronal Interact. 2002; 2:270–273. 15758451

[2] Nye HL, Cameron JA, Chernoff EA, Stocum DL. Regeneration of the urodele limb: a review. Dev Dyn. 2003; 226: 280–294. 12557206 10.1002/dvdy.10236

[3] Moseley FL, Faircloth ME, Lockwood W, Marber MS, Bicknell KA, Valasek G, et al Limitations of the MRL mouse as a model for cardiac regeneration. J Pharm Pharmacol.2011; 63: 648–656. 10.1111/j.2042-7158.2011.01261.x 21492166

[4] Li C. Deer antler regeneration: a stem cell-based epimorphic process. Birth Defects Res C Embryo Today.2012; 96: 51–62. 10.1002/bdrc.21000 22457177

[5] Li C. Histogenetic aspects of deer antler development. Front Biosci (Elite Ed). 2013; 5: 479–489. 23277003 10.2741/e629

[6] Li C, Yang F, Li G, Gao X, Xing X, Deng X, et al Antler regeneration: a dependent process of stem tissue primed via interaction with its enveloping skin. J Exp Zool A Ecol Genet Physiol. 2007; 307: 95–105. 17177282 10.1002/jez.a.352

[7] Li C, Zhao H, Liu Z, McMahon C. Deer antler—A novel model for studying organ regeneration in mammals. Int J Biochem Cell Biol. 2014; 56C: 111–122. 10.1016/j.biocel.2014.07.007 25046387

[8] Li C, Yang F, Sheppard A. Adult stem cells and mammalian epimorphic regeneration-insights from studying annual renewal of deer antlers. Curr Stem Cell Res Ther.2009; 4: 237–251. 19492976 10.2174/157488809789057446

[9] Li C, Harper A, Puddick J, Wang W, McMahon C. Proteomes and signalling pathways of antler stem cells. PLoS One.2012; 7: e30026 10.1371/journal.pone.0030026 22279561 PMC3261186

[10] Kierdorf U, Kierdorf H, Szuwart T. Deer antler regeneration: cells, concepts, and controversies. J Morphol. 2007; 268: 726–738. 17538973 10.1002/jmor.10546

[11] Heber-Katz E, Zhang Y, Bedelbaeva K, Song F, Chen X, Stocum DL. Cell cycle regulation and regeneration. Curr Top Microbiol Immunol. 2013; 367: 253–276. 10.1007/82_2012_294 23263201

[12] Arima Y, Hirota T, Bronner C, Mousli M, Fujiwara T, Niwa S, et al Down-regulation of nuclear protein ICBP90 by p53/p21Cip1/WAF1-dependent DNA-damage checkpoint signals contributes to cell cycle arrest at G1/S transition. Genes Cells. 2004; 9(2): 131–142. 15009091 10.1111/j.1356-9597.2004.00710.x

[13] Hartwell L H, Kastan M B. Cell cycle control and cancer. Science. 1994; 266 (5192): 1821–1828. 7997877 10.1126/science.7997877

[14] Hartwell L H, Weinert T A. Checkpoints: controls that ensure the order of cell cycle events. Science. 1989; 246: 629–634. 2683079 10.1126/science.2683079

[15] Bedelbaeva K, Snyder A, Gourevitch D, Clark L, Zhang XM, Leferovich J, et al Lack of p21 expression links cell cycle control and appendage regeneration in mice. Proc Natl Acad Sci U S A. 2010; 107: 5845–5850. 10.1073/pnas.1000830107 20231440 PMC2851923

[16] Liu J, Zhang C, Hu W, Feng Z. Tumor suppressor p53 and its mutants in cancer metabolism. Cancer Lett. 2015; 356: 197–203. 10.1016/j.canlet.2013.12.025 24374014 PMC4069248

[17] Zhang L, Mei Y, Fu NY, Guan L, Xie W, Liu HH, et al TRIM39 regulates cell cycle progression and DNA damage responses via stabilizing p21. Proc Natl Acad Sci U S A. 2012; 109: 20937–20942. 10.1073/pnas.1214156110 23213251 PMC3529087

[18] Waga S, Hannon G J, Beach D, Stillman B. The p21 inhibitor of cyclin-dependent kinases controls DNA replication by interaction with PCNA. Nature. 1994; 369 (6481): 574–578 7911228 10.1038/369574a0

[19] Clark LD, Clark RK, Heber-Katz E. A new murine model for mammalian wound repair and regeneration. Clin Immunol Immunopathol. 1998; 88: 35–45. 9683548 10.1006/clin.1998.4519

[20] Arthur LM, Heber-Katz E. The role of p21 in regulating mammalian regeneration. Stem Cell Res Ther. 2011; 2: 30 10.1186/scrt71 21722344 PMC3152998

[21] Sun H, Yang F, Chu W, Zhao H, McMahon C, Li C. Lentiviral-mediated RNAi knockdown of Cbfa1 gene inhibits endochondral ossification of antler stem cells in micromass culture. PLoS One. 2012; 7: e47367 10.1371/journal.pone.0047367 23056636 PMC3467256

[22] Jonathan B, Richard G V. Antennal expressed genes of the yellow fever mosquito (Aedes aegypti L.) characterization of odorant-binding protein 10 and takeout. Insect Biochem Mol Biol. 2005; 35: 961–979. 15978998 10.1016/j.ibmb.2005.03.010

[23] Chu W, Zhao H, Yang F, Xing X, Liu G. Construction and characterisation of lentiviral vector of RNA interference of collagen type X of sika deer. Journal of China Agricultural University. 2009; 14: 29–34.

[24] Twentyman PR, Luscombe M. A study of some variables in a tetrazolium dye (MTT) based assay for cell growth and chemosensitivity. Br J Cancer. 1987; 56: 279–285. 3663476 10.1038/bjc.1987.190 PMC2002206

[25] Andrighetti-Frohner CR, Kratz JM, Antonio RV, Creczynski-Pasa TB, Barardi CR, Simoes CM. In vitro testing for genotoxicity of violacein assessed by Comet and Micronucleus assays. Mutat Res.2006; 603: 97–103. 16359912 10.1016/j.mrgentox.2005.11.001

[26] Zhou Z, Wang C, Liu H, Huang Q, Wang M, Lei Y (2013) Cadmium induced cell apoptosis, DNA damage, decreased DNA repair capacity, and genomic instability during malignant transformation of human bronchial epithelial cells. Int J Med Sci. 2013; 10: 1485–1496. 10.7150/ijms.6308 24046522 PMC3775105

[27] Chaffey GS, Lloyd DJ, Skeldon AC, Kirkby NF. The effect of the G1-S transition checkpoint on an age structured cell cycle model. PLoS One. 2014; 9: e83477 10.1371/journal.pone.0083477 24416166 PMC3886982

[28] Schmidt T, David CN. Gland cells in Hydra: cell cycle kinetics and development. J Cell Sci. 1986; 85: 197–215. 3539952 10.1242/jcs.85.1.197

[29] Stocum DL, Cameron JA. Looking proximally and distally: 100 years of limb regeneration and beyond. Dev Dyn.2011; 240: 943–968. 10.1002/dvdy.22553 21290477

[30] Celton-Morizur S, Desdouets C. Polyploidization of liver cells. Adv Exp Med Biol. 2010; 676: 123–135. 20687473 10.1007/978-1-4419-6199-0_8

[31] Michalopoulos GK, DeFrances MC. Liver regeneration. Science. 1997; 276: 60–66. 9082986 10.1126/science.276.5309.60

[32] Murphy ED, Roths JB. A Y chromosome associated factor in strain BXSB producing accelerated autoimmunity and lymphoproliferation. Arthritis Rheum. 1979; 22: 1188–1194. 315777 10.1002/art.1780221105

[33] Gulappa T, Reddy RS, Suman S, Nyakeriga AM, Damodaran C. Molecular interplay between cdk4 and p21 dictates G0/G1 cell cycle arrest in prostate cancer cells. Cancer Lett. 2013; 337: 177–183. 10.1016/j.canlet.2013.05.014 23684928 PMC3752915

[34] Barnum KJ, O'Connell MJ. Cell cycle regulation by checkpoints. Methods Mol Biol. 2014; 1170: 29–40. 10.1007/978-1-4939-0888-2_2 24906307 PMC4990352

[35] Gotz C, Montenarh M. p53: DNA damage, DNA repair, and apoptosis. Rev Physiol Biochem Pharmacol. 1996; 127: 65–95. 8533012 10.1007/BFb0048265

[36] Kaina B. DNA damage-triggered apoptosis: critical role of DNA repair, double-strand breaks, cell proliferation and signaling. Biochem Pharmacol. 2003; 66: 1547–1554. 14555233 10.1016/s0006-2952(03)00510-0

[37] Michieli P, Chedid M, Lin D, Pierce JH, Mercer WE, Weng MS. Induction of WAF1/CIP1 by a p53-independent pathway. Cancer Res. 1994; 54: 3391–3395. 8012956

[38] Gorgoulis VG, Vassiliou LV, Karakaidos P, Zacharatos P, Kotsinas A, Liloglou T, et al Activation of the DNA damage checkpoint and genomic instability in human precancerous lesions. Nature. 2005; 434: 907–913. 15829965 10.1038/nature03485

[39] Perucca P, Cazzalini O, Madine M, Savio M, Laskey RA, Vinnini V, et al Loss of p21 CDKN1A impairs entry to quiescence and activates a DNA damage response in normal fibroblasts induced to quiescence. Cell Cycle. 2009; 8: 105–114. 19106607 10.4161/cc.8.1.7507

[40] Martinez LA, Yang J, Vazquez ES, Rodriguez-Vargas Mdel C, Olive M, Hsieh JT, et al p21 modulates threshold of apoptosis induced by DNA-damage and growth factor withdrawal in prostate cancer cells. Carcinogenesis. 2002; 23: 1289–1296. 12151346 10.1093/carcin/23.8.1289

[41] Abbas T, Dutta A. p21 in cancer: intricate networks and multiple activities. Nat Rev Cancer. 2009; 9: 400–414. 10.1038/nrc2657 19440234 PMC2722839

[42] Basu N, Saha S, Khan I, Ramachandra SG, Visweswariah SS. Intestinal cell proliferation and senescence are regulated by receptor guanylyl cyclase C and p21. J Biol Chem. 2014; 289: 581–593. 10.1074/jbc.M113.511311 24217248 PMC3879579

[43] Brown J P, Wei W, Sedivy J M. Bypass of senescence after disruption of p21CIP1/WAF1 gene in normal diploid human fibroblasts. Science. 1997; 277(5327):831–834. 9242615 10.1126/science.277.5327.831

[44] Choudhury A R, Ju Z, Djojosubroto M W, Schienke A, Lechel A, Schaetzlein S. Cdkn1a deletion improves stem cell function and lifespan of mice with dysfunctional telomeres without accelerating cancer formation. Nat Genet. 2007; 39(1): 99–105. 17143283 10.1038/ng1937

[45] Haferkamp S, Tran S L, Becker T M, Scurr L L, Kefford R F, Rizos H. The relative contributions of the p53 and pRb pathways in oncogene-induced melanocyte senescence. Aging (Albany NY). 2009; 1(6):542–556. 20157537 10.18632/aging.100051 PMC2806033

